# HeiPorSPECTRAL - the Heidelberg Porcine HyperSPECTRAL Imaging Dataset of 20 Physiological Organs

**DOI:** 10.1038/s41597-023-02315-8

**Published:** 2023-06-24

**Authors:** Alexander Studier-Fischer, Silvia Seidlitz, Jan Sellner, Marc Bressan, Berkin Özdemir, Leonardo Ayala, Jan Odenthal, Samuel Knoedler, Karl-Friedrich Kowalewski, Caelan Max Haney, Gabriel Salg, Maximilian Dietrich, Hannes Kenngott, Ines Gockel, Thilo Hackert, Beat Peter Müller-Stich, Lena Maier-Hein, Felix Nickel

**Affiliations:** 1 Department of General, Visceral, and Transplantation Surgery, Heidelberg University Hospital, Heidelberg, Germany; 2 Division of Intelligent Medical Systems, German Cancer Research Center (DKFZ), Heidelberg, Germany; 3 Faculty of Mathematics and Computer Science, Heidelberg University, Heidelberg, Germany; 4 HIDSS4Health – Helmholtz Information and Data Science School for Health, Karlsruhe, Heidelberg, Germany; 5 National Center for Tumor Diseases (NCT) Heidelberg, a partnership between DKFZ and Heidelberg University Hospital, Heidelberg, Germany; 6 Medical Faculty, Heidelberg University, Heidelberg, Germany; 7 Division of Plastic Surgery, Department of Surgery, Brigham and Women’s Hospital, Harvard Medical School, Boston, MA USA; 8 Department of Urology, Medical Faculty of Mannheim at the University of Heidelberg, Mannheim, Germany; 9 Department of Anesthesiology, Heidelberg University Hospital, Heidelberg, Germany; 10 Department of Visceral, Transplant, Thoracic and Vascular Surgery, Leipzig University Hospital, Leipzig, Germany; 11 Department of General, Visceral, and Thoracic Surgery, University Hospital Hamburg-Eppendorf, Hamburg, Germany

**Keywords:** Preclinical research, Translational research

## Abstract

Hyperspectral Imaging (HSI) is a relatively new medical imaging modality that exploits an area of diagnostic potential formerly untouched. Although exploratory translational and clinical studies exist, no surgical HSI datasets are openly accessible to the general scientific community. To address this bottleneck, this publication releases HeiPorSPECTRAL (https://www.heiporspectral.org; 10.5281/zenodo.7737674), the first annotated high-quality standardized surgical HSI dataset. It comprises 5,758 spectral images acquired with the TIVITA^®^ Tissue and annotated with 20 physiological porcine organs from 8 pigs per organ distributed over a total number of 11 pigs. Each HSI image features a resolution of 480 × 640 pixels acquired over the 500–1000 nm wavelength range. The acquisition protocol has been designed such that the variability of organ spectra as a function of several parameters including the camera angle and the individual can be assessed. A comprehensive technical validation confirmed both the quality of the raw data and the annotations. We envision potential reuse within this dataset, but also its reuse as baseline data for future research questions outside this dataset.

**Table:** 

Measurement(s)	Spectral Reflectance
Technology Type(s)	Hyperspectral Imaging
Sample Characteristic - Organism	*Sus scrofa*

## Background & Summary

Spectral imaging is one of the evolving attractive surgical imaging techniques^1^. While conventional endoscopy is based on electromagnetic wavelengths in the visible spectrum and features relatively low information content, Hyperspectral Imaging (HSI) can cover a much broader wavelength range at much higher spectral resolution. Having successfully been used in multiple industries, it is now also finding its way into healthcare through certified medical devices^2,3^. Objective evaluation of tissue oxygenation and perfusion^4–7^, the identification of malignancy^8^, inflammation or sepsis^9^ as well as computer-assisted decision-making^1^ are examples of potential future clinical applications include the.

Although exploratory translational and clinical studies^6,10–24^ and few public datasets exist outside the field of surgery^25,26^, no standardized or annotated surgical HSI datasets of multiple physiological organs and tissues of a well-described collective are accessible to the general scientific community. To the best of our knowledge, the largest currently publicly available medical HSI datasets are the ODSI-DB dataset (composed of 316 images from 30 patients regarding oral and dental samples)^26^ and the HELICoiD dataset (composed of 36 images from 22 patients regarding tumorous and healthy brain tissue)^25^. Our dataset substantially surpasses these in volume (5756 images) as well as constitutes the first open source HSI dataset in the application domain of visceral surgery (e.g., for organ discrimination).

With the mission to support further research and advancement of surgical HSI as a diagnostic tool and beyond, this paper presents the open **Hei**delberg **Por**cine Hyper**SPECTRAL** Imaging Dataset (**HeiPorSPECTRAL;** https://www.heiporspectral.org; 10.5281/zenodo.7737674)^27^. This dataset includes HSI data of 20 physiological organs and tissues in 8 *in-vivo* pig models per organ. Recordings were obtained in a total of 11 pig models as not every organ could be recorded in every animal. This was due to several inherent challenges in the pig model (e.g., organ aplasia, organ adhesions from presumably previous trauma, circulation insufficiency after several hours of narcosis, etc.). Images were repetitively taken three times (“repetition”) from three different angles (“angle”) as well as four intraoperative situations (“situs”) (36 recordings per organ and pig) (Fig. 2).

The reuse potential of this dataset in future applications is manifold: Firstly, one could further expand the existing work^28,29^ on intraoperative tissue differentiation and characterization based on this dataset. Secondly, this dataset can be used as a baseline for comparison with other HSI datasets that for example include different species or pathological states such as tissue malperfusion, inflammation or malignancy.

Of note, currently, there appears to be a tendency of many individual, independent research groups publishing exploratory and proof-of-concept HSI studies with small numbers of experimental animals. This is mainly due to the high-interest nature of publishing about innovative technologies that can be applied to a variety of scenarios such as multiple organ systems. However, there are several main problems with this current situation:

1. **Dissipation of resources**: Research groups do not share HSI data and therefore might record similar data. For example, if multiple liver research projects are conducted and one group focuses on portal vein occlusion while the other focuses on inflammation or fibrosis, chances are high that data required by both, e.g. the spectral reflectance of healthy liver, might be recorded multiple times, although one high-quality recording could serve multiple research questions^30,31^. This represents avoidable time and monetary burdens.
2. **Unnecessary baseline data**: Often this commonly required data will be baseline data, e.g. of physiological organs. Available animal numbers could therefore instead be invested in additional measurements of non-baseline groups or, alternatively, the total number of animals needed per study could be reduced.
3. **Ethical challenges**: As a result from this situation, basic principles of humane experimental technique such as “replace, reduce, refine” are not adhered to^32^ and optimization is needed from an ethical standpoint.
4. **Unexploited potential**: Many of the experimental proof-of-concept HSI studies with clinical focus feature numbers of 5 to 20 pigs per group^6,14,33,34^. This is sufficient for exploratory and descriptive studies, but not for machine-learning applications. For instance, it could already be shown that with a large enough sample size (i.e. 46 pigs), reflectance spectra are highly organ-specific and the prediction accuracy of 20 organ classes could be as high as 95% and more^28^. It therefore seems plausible that results such as a recognition sensitivity of, e.g. only 79.0% and 86.0% for 3 organ classes^34^ might be due to insufficiently small numbers of animals, which do not allow for successful training, validation and testing of machine-learning algorithms. Therefore, applications with actual potential for clinical use could falsely be discarded at early stages due to perceived imprecision caused solely by the study setup.
5. **Lack of external validation of Machine Learning algorithms**: Most of the currently existing publications on the combination of HSI and machine learning do not report results on external datasets^35^. As a consequence, subsequent application of published machine learning algorithms often disappoints in accuracy on external data – by now a well-known pitfall in artificial intelligence^36^.

These arguments depict the necessity for a publicly available HSI database resource of porcine organs and tissues that other research groups can use to base their experiments upon or employ for external validation. Making this dataset publicly accessible aims to support future research and advancement of medical HSI as a diagnostic tool and beyond.

An extensive visual comparison of spectral signatures and differences between these 20 classes as well as more detailed hyperspectral profile visualizations of the different organs and tissues can be found in the repository (https://figures.heiporspectral.org/label_profiles/). The minimal effects “situs”, “angle” and “repetition” have on the spectral reflectance can also be found in cited literature^28^.

With **HeiPorSPECTRAL**^27^ (Fig. 2), we release an HSI data set of unprecedented size that addresses all of these issues. In publishing this dataset, we aim to support future research towards HSI as a powerful diagnostic tool in clinical medicine and surgery.

## Methods

### Hyperspectral imaging

HSI data was acquired using the TIVITA® Tissue system from Diaspective Vision GmbH, which is the first commercially available, medically certified surgical HSI camera. The application of HSI in real-world surgeries poses several requirements regarding the choice of the device: (1) The device needs to be operable in a way such that sterility requirements are met. (2) The device should be medically certified for use in surgeries. (3) Not only the computed parameter images, but also the raw HSI cubes need to be exportable. To the best of our knowledge, the TIVITA® system from Diaspective Vision is the only currently available HSI system meeting all three requirements. It was therefore decided to use the TIVITA® system in this porcine study since the data should serve as a baseline for future studies in humans and a domain gap caused by a change in device would not be desirable.

The TIVITA® HSI camera functions as a pushbroom spectrometer device: Using a moving slit, the image is scanned line by line. Each line is spectrally dispersed onto a CMOS sensor and spectral channels are generated by binning the signal from specific sets of pixels.

This results in 100 non-overlapping spectral channels between 500 and 1000 nm with a width of approximately 5 nm each. The camera records reflectance in arbitrary units and provides a high spectral resolution in the visible as well as near-infrared range. The final three-dimensional datacube has a spatial resolution of 480 × 640 pixels and 100 spectral channels with a bandwidth of 5 nm per step along the third dimension (λ) with a total of 30,720,000 reflectance values in arbitrary units.

The original spectral raw data is stored as a.dat file by the camera software, which is an open data format provided by the camera manufacturer. Of all hyperspectral data formats, it is currently the most widely used in medical HSI^37^. Details on how to read, process and convert these.dat hypercubes is provided in the documentation on the repository homepage. Certain aspects of relevant metadata such as the characteristics of the spectral channels are not stored together with the HSI raw data, but are provided in the repository as they hold for the entire dataset.

Standardized illumination is provided by six integrated halogen lights. All other light sources were switched off during measurements to prevent straylight. Illumination uniformity was balanced out using dark subtraction and white balancing before every surgery. The HSI cubes stored in the.dat files are already preprocessed with this dark subtraction and white balancing and thus contain estimated reflectances. White balancing was not adapted to non-fronto-parallel tissue situations as it cannot be performed for any arbitrary acquisition angle during real-world surgery due to sterility and time constraints and although this study focused on a research setting, we aimed to take future real-world applications into consideration.

**Fig. 1:**
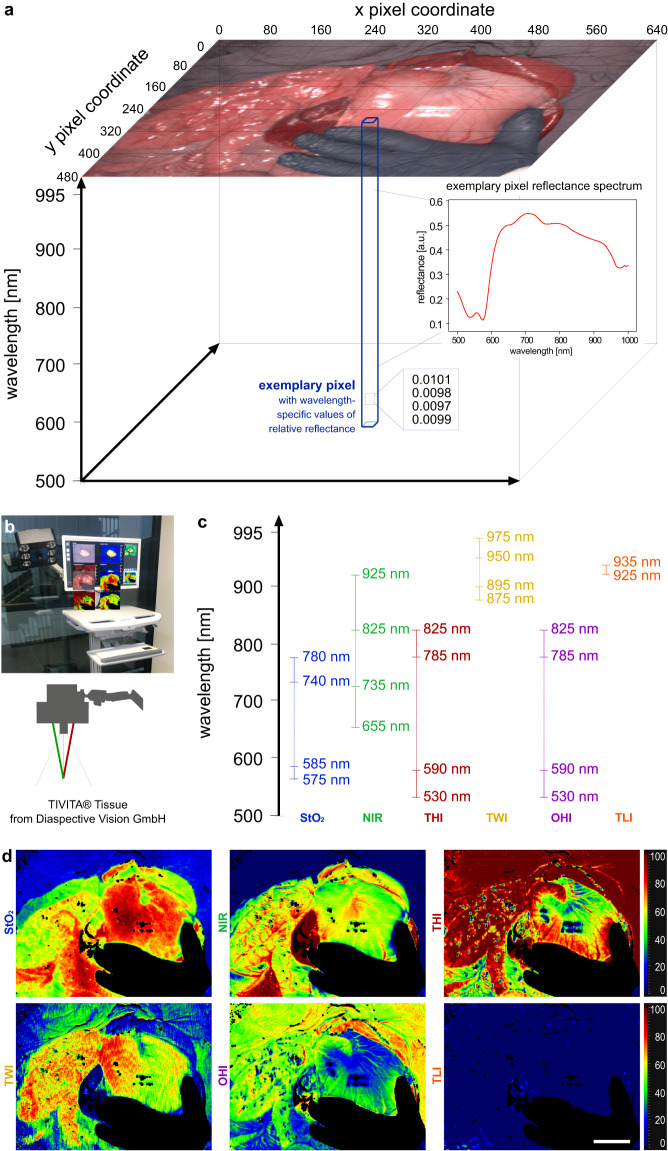
Hyperspectral data cube and camera system. (**a**) visualization of a three-dimensional hyperspectral datacube. (**b**) TIVITA® Tissue hyperspectral camera system from Diaspective Vision GmbH; the red-and-green laser light targeting system is schematically indicated. (**c**) wavelength areas of index pictures. (**d**) color-coded index pictures.

The distance of the objective to the specimen is standardized via a red-and-green laser light targeting system as indicated in Fig. 1 to remain at 50 cm. This works through the projection of two LED beams from the camera head from two different angles meeting at 50 cm distance from the objective on the specimen. A light power of 0.22 Watt at the imaging distance of 50 cm was measured using a thermal power sensor (S425C-L, Thorlabs). The distance of 50 cm in combination with the recorded number of pixels results in a spatial resolution of approximately 0.45 mm/pixel. The field of view is 20.5 × 27.5 cm. This resolution is sufficient for evaluating global processes within the entirety of an organ (e.g. perfusion). However, resolution will be too low to investigate near-microscopic heterogeneity within the organs.

The camera software calculates several color-coded functional tissue parameters visualized through index pictures including the tissue oxygenation index (StO_2_), the near-infrared perfusion index (NIR), the tissue hemoglobin index (THI), the tissue water index (TWI), the organ hemoglobin index (OHI) and the tissue lipid index (TLI). Also, RGB images were reconstructed using the TIVITA® proprietary software. While their underlying formulas, calculations and involved wavelengths can be reviewed in cited literature^38^, the parameter details are confidential and the TIVITA® software is proprietary. Consequently, reproducing the index values is not directly possible. Therefore, the index values for every image are provided as numerical data as well as color-coded index images. The rest of the data, i.e. the spectral reflectance curves, can be completely reproduced and used for subsequent analyses with the code developed and provided on the repository and GitHub. The TIVITA® software is therefore not required to use the data. A schematic overview of the technology can be found in Fig. 1. More elaborate description of the technology can be found in the literature^28,38–40^.

HSI systems generally suffer from a trade-off between spectral, spatial and temporal resolution: Snapshot sensors sacrifice spectral and spatial resolution for temporal resolution, wavelength-scanning systems (e.g., by use of filter wheels or electronically tunable filters) offer high spatial resolution, but limited spectral and temporal resolution, and pushbroom devices, such as the TIVITA® camera, offer high spectral resolution at the cost of spatial and temporal resolution. Therefore, structures that are smaller than the spatial resolution limit (e.g., capillaries) cannot be distinguished on this HSI setup. Consequently, the benefit of large spatial measurement areas as in this study comes at the cost of potentially missing diagnostically critical heterogeneity such as fibrosis or steatosis existing within many organs due to the relatively low resolution. The spectral heterogeneity across pixels within one organ is not displayed in the manuscript as the primary analysis is based on the median spectra computed over each organ annotation. To close this gap, there are visualizations on the repository that show the median spectra per annotation together with the corresponding standard deviation across pixels (https://figures.heiporspectral.org/view_organs/01_stomach/P086%232021_04_15_11_48_06.html?nav=show&link_index=20).

### Experimental models and surgical procedure

The experiments in *in-vivo* porcine models were approved by the Committee on Animal Experimentation of the regional council Baden-Württemberg in Karlsruhe (G-161/18 and G-262/19). All experimental animals were managed according to the directives of the European Community Council (2010/63/EU), ARRIVE guidelines^41^ and according to German laws for animal use and care. Pigs (“*Sus scrofa ssp. Domesticus*”) with an ordered weight of 35.0 kg were used as the experimental model (approximately 50% male, 50% female). Pharmaceutical treatment and narcosis were performed according to institution standards as described in earlier publications^7,28^.

Pharmacological calculations were adapted to body weight and generalized for a 40 kg pig. Intramuscular injection of the neuroleptic azaperone (Stresnil® 40 mg/ml by Elanco®) with 6 mg/kg (≈ 6 ml = 240 mg) was used for initial sedation. Intramuscular injection of a combination of midazolam (Midazolam-hameln® 5 mg/ml by hameln pharma plus gmbh®) with 0,75 mg/kg (≈ 6 ml = 30 mg) and ketamine (Ketamin 10%® by Heinrich Fromme®) with 10 mg/kg (≈4 ml = 400 mg) was applied to establish analgosedation. This was followed by intubation and pressure-controlled ventilation with a minimal alveolar concentration (MAC) of 1,1 under sevoflurane®. Intraoperative narcosis was established through a combined narcosis with sevoflurane®, intravenous 0,2 mg/kg/h midazolam (≈1,5 ml/h = 7,5 mg/h) and 8,75 mg/kg/h ketamine (≈3,5 ml/h = 350 mg/h) at a rate of 5 ml/h. Body temperature was maintained with electrically controlled heat blankets. No relaxant agents were applied.

In order to ensure validity of the porcine *in-vivo* model, pigs were extensively monitored intraoperatively as respiratory and cardiocirculatory insufficiency would affect the spectral measurements due to hemoglobin being the main tissue chromophore. Monitoring included electrocardiography, body core temperature sensors, invasive arterial blood pressure sensors, pulse oximetry and central venous pressure sensors. HSI data was only recorded in case of normofrequent sinus rhythm with a body core temperature above 36 °C, sufficient mean arterial blood pressure, and oxygenation of at least 90%.

After median laparotomy and thoracotomy all relevant organs and tissues were mobilized while strictly protecting their vascular supply. Recordings were conducted for each of the 20 organs and tissues in the following fashion and resulting in a total of about 36 recordings for each organ:

- in 8 pigs per organ (11 pigs in total)
- with 4 situs: different intraoperative positionings of the recorded organ
- from 3 angles: perpendicular to the organ surface (angle 1: 0°) as well as approximately 25° from the anatomical left (angle 2: −25°) and 25° from the anatomical right (angle 3: +25°)
- with 3 repetitions of exactly the same situation

**Fig. 2:**
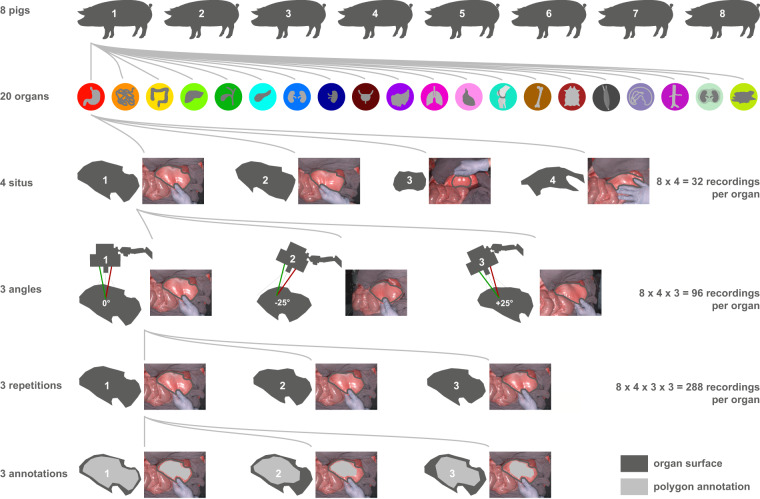
Schematic visualization of the recording and annotation protocol. (**a**) every organ was recorded in 8 pigs at 4 different regions (“situs”), from 3 different angles with 3 recordings/repetitions each. Annotations were done independently by 3 different annotators using a polygon tool.

**Fig. 3:**
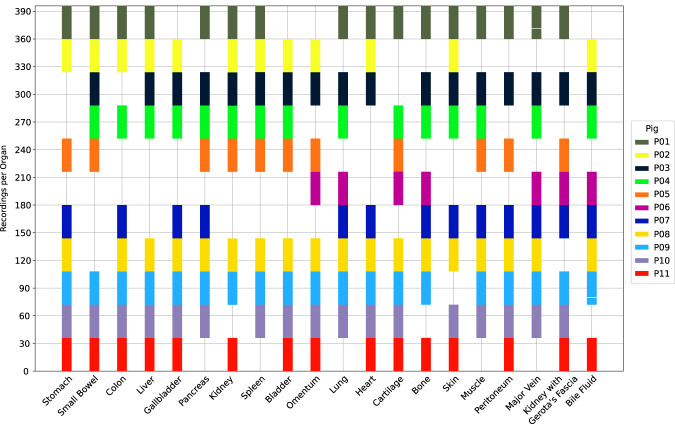
Dataset visualization. Overview of the distribution of recordings in 8 pigs per organ across 11 pigs. Each bar indicates the block of images which were recorded for that organ and pig. One recording for “major vein” and one for “bile fluid” is missing in P01 and P09 (indicated with a white line in the bar).

A graphical abstract of the data acquisition protocol can be found in Fig. 2. Data of a total number of 11 animals was included in the analysis as not all organs could be recorded in the same 8 pigs (e.g. due to premature exitus due to cardiocirculatory and respiratory insufficiency during the experiments) (Fig. 3).

After surgery, pigs were euthanized with a rapid intravenous application of 50 ml of potassium chloride solution. Death was pronounced upon an end-expiratory CO_2_ partial pressure below 8 mmHg.

## Data Records

**HeiPorSPECTRAL**^27^ is a standardized data subset of an HSI dataset with extensive analysis published earlier^28^. The 11 pigs contributing to this data subset are referred to as P01 to P11 throughout this manuscript. An overview of the distribution of recordings in 8 pigs per organ across 11 pigs can be found in Fig. 3. It is important to mention that for the label “major vein” and “bile fluid”, 1 of the intended 36 recordings of the standardized recording protocol is missing in P01 and P09 due to technical difficulties during the recording process. Every annotated region exclusively contains physiological tissue and this is also true for all unannotated regions except for a few images that are explicitly specified and explained in the FAQs on the repository website.

### Data annotation

In each acquired organ image series, representative image regions of the following structures depending on the respective organ image series were annotated: stomach, small bowel, colon, liver, gallbladder, pancreas, kidney, spleen, bladder, omentum, lung, heart, cartilage, bone, skin, muscle, peritoneum, major vein, kidney with Gerota’s fascia and bile fluid. Polygon annotations were performed by three independent medical experts (annotator 1–3) using the HyperGui tool (https://github.com/MIC-Surgery-Heidelberg/HyperGui). In order to obtain high-quality annotations, the following instructions were given to the annotators: Polygon areas should omit marginal areas, superficial blood vessels and fat as well as artefacts such as tissue deformation, shaded areas, contamination with dyes or body fluids such as bile fluid, previous manipulation such as contusion or abrasion, and possible impairment of perfusion such as thrombosis. The regions should include only highly representative physiological areas; therefore, it is guaranteed that analyzed pixels were always representative of the organ label. Consequently, there are additional adjacent pixels that could have been selected as well, but were not selected based on the judgment of the annotator and the premise to not include non-physiological or non-representative tissue spectra under any circumstance. In case of several possible regions that are separated by any of the aforementioned artefacts, the largest possible should be annotated.

### Data preprocessing

To estimate spectral reflectance from the measured spectral power distributions, dark subtraction and white balancing were performed prior to every surgery *approximately* orthogonally to the surface. Since this step is directly performed by the camera software, the HSI cubes stored in the.dat files already contain estimated reflectances. Although variations in the imaging distance are limited due to an integrated distance calibration system, small distance variations still lead to undesired variations in the scaling of the measured reflectance spectra. To remove such effects and improve the comparability of reflectance spectra across images and devices, the HSI cubes were further preprocessed with L1-normalization such that for every image pixel the 100 corresponding absolute reflectance values sum up to 1.

### Data visualization with PCA and UMAP

**Fig. 4:**
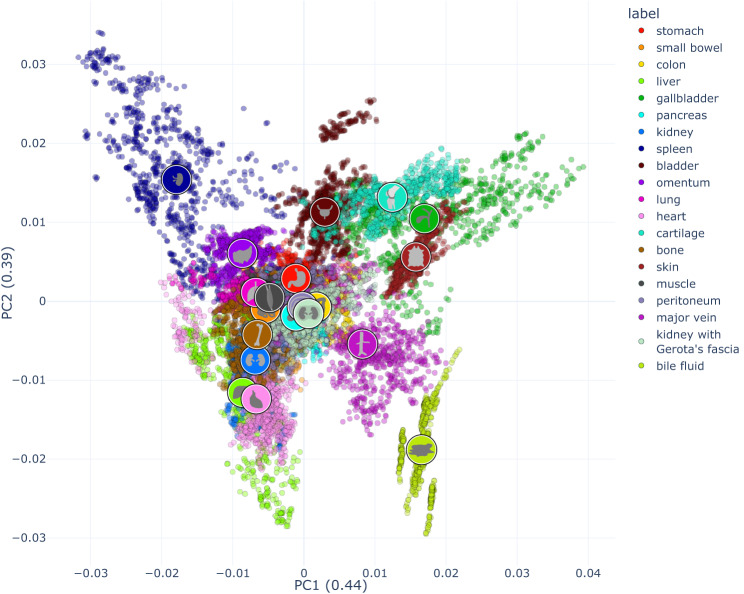
Visualization of spectral similarity with PCA as a linear multi-dimensionality reduction tool with a cumulative explained variance of 0.83 (0.44 for x; 0.39 for y). Each point represents the median spectrum across one annotation of one organ in one image of one pig. The organ symbols are placed at the centroid of the corresponding organ distribution. An interactive version of this figure can be found online (https://figures.heiporspectral.org/paper_figures/Figure_04_PCA.html).

**Fig. 5:**
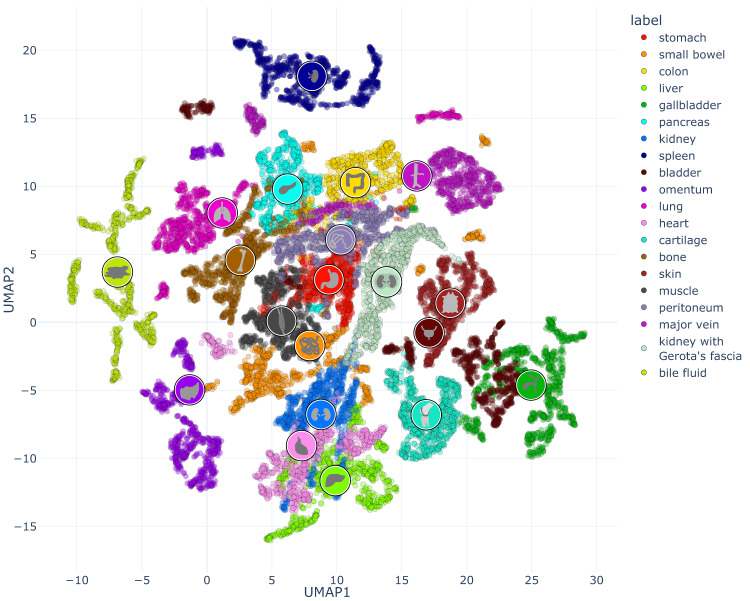
Visualization of spectral similarity with UMAP as a non-linear multi-dimensionality reduction tool. Each point represents the median spectrum across one annotation of one organ in one image of one pig of one annotation. The organ symbols are placed at the centroid of the corresponding organ distribution. An interactive version of this figure can be found and online (https://figures.heiporspectral.org/paper_figures/Figure_05_UMAP.html). A supervised version of the UMAP projection can be found online (https://figures.heiporspectral.org/paper_figures/Supplemental_Figure_09_UMAP_supervised.html).

**Fig. 6:**
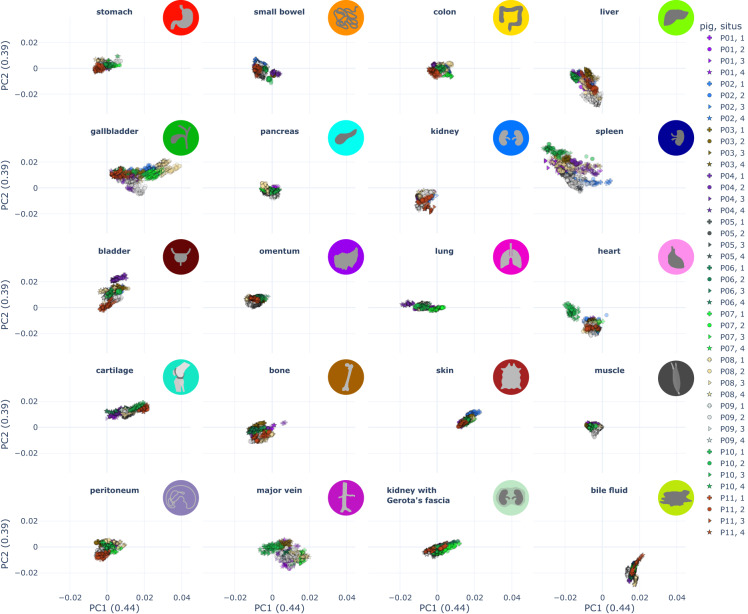
Visualization of spectral similarity with PCA stratified by organ; each point represents the median spectrum across one annotation of one organ in one image of one pig. Different shapes represent different situs. Different colors represent 11 different pigs. An interactive version of this figure which allows selecting the color and shape coding of the different attributes (pig, annotation, situs, angle, repetition) can be found online (https://figures.heiporspectral.org/paper_figures/Figure_06_PCA_single_organs.html).

For enhanced visualization of the multi-dimensional dataset, dimensionality reduction techniques were applied. The dataset was processed in that for each annotation one characteristic reflectance spectrum was obtained by calculating the median spectra from the L1-normalized spectra of all pixels in the annotated region. Therefore, each of the data points in the depictions (Figs. 4–6**)** resembles the reflectance of a single annotation of one organ in one image of one pig.

Principal Component Analysis (PCA)^42^ (Fig. 4) and Uniform Manifold Approximation and Projection (UMAP)^43–45^ (Fig. 5) were applied on this preprocessed data (median of L1-normalized pixel spectra).

UMAP was used for dimensionality reduction for several reasons. It is a machine learning method commonly used to enable a low-dimensional visualization by reducing the number of dimensions of high-dimensional data of the reflectance spectra in an unsupervised fashion. By providing non-linear low-dimensional embeddings of high‐dimensional data, UMAP can preserve a greater degree of local neighborhoods from the high-dimensional space compared to linear methods such as PCA^42^ and Linear discriminant analysis (LDA)^46^. UMAP parameters were evaluated by visual inspection yielding a local neighborhood size of 50 and a minimal distance of 0.8.

For the PCA visualizations stratified by organ (Fig. 6), the corresponding organ spectra were transformed using the existing transformation matrix computed on the full dataset. This ensured that data points in Figs. 4, 6 are comparable as they belong to the same coordinate system.

The PCA showed distinct clustering of the spectral recordings of the different organ and tissue entities with a cumulative explained variance of 0.83 (Fig. 4).

The UMAP as a non-linear multi-dimensionality reduction tool showed even more distinctive clustering (Fig. 5). It can be seen that organs such as “spleen” and “bile fluid” form isolated clusters while other organs such as “kidney with Gerota’s fascia” and “peritoneum” greatly overlap.

## Technical Validation

In our technical validation we assessed the quality of the HSI data, the quality of the annotations and the quality of the animal model, in order to guarantee integrity of data.

**Fig. 7:**
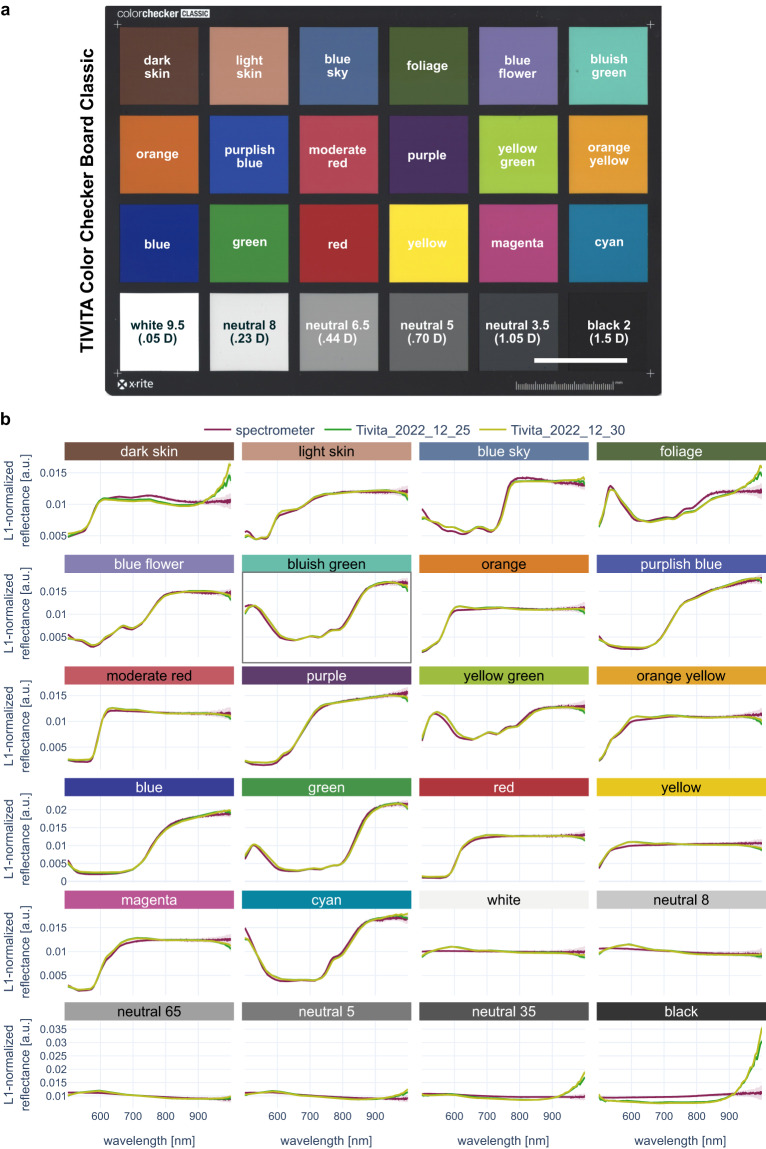
A checkerboard-based technical validation confirms very good reproducibility and high validity of the HSI images. (**a**) Reconstructed RGB image from a hyperspectral imaging (HSI) recording of a colorchecker board. (**b**) Spectrometer (purple) and HSI (light and dark green) recordings for the 24 different color chips. The median spectrum across all available recordings (100 recordings for spectrometer, 13 recordings for HSI) is denoted by a solid line, the corresponding standard deviation as a shaded area. An interactive version of this figure can be found and online (https://figures.heiporspectral.org/paper_figures/Figure_07_colour_checker_L1.html).
  Although variations in the imaging distance are limited due to an integrated distance calibration system, small distance variations still lead to undesired variations in the scaling of the measured reflectance spectra. Such scaling differences are especially magnified when comparing reflectance spectra measured with different devices, e.g. the HSI camera system versus a spectrometer. Figure 8 shows the same data as Fig. 7, but prior to L1-normalization. L1-normalization largely improves the comparability of reflectance spectra in such cases. Figure 8 for example shows the same reflectance spectra as in Fig. 7, but without L1-normalization. It can be seen that the former congruence between the spectrometer and the HSI camera is no longer evident and based on the unnormalized reflectance spectra, a judgment whether HSI and spectrometer measurements are in agreement is hardly possible. This allows for the conclusion that normalization is a recommendable step in order to obtain comparability between spectral reflectance measurements.

1. **Quality of the animal model:** In order to visualize a possible hidden heterogeneity between the recorded individuals, PCA was performed at organ level (Fig. 6). Although some minor clustering could be observed, e.g. for heart or muscle, the different subjects contributed only little to the overall variability (Fig. 4). This has been quantitatively been confirmed by a mixed model analysis that was previously performed on a dataset of 46 pigs, including this dataset. Here it could be shown that inter-pig variability only accounts for 2.3% of the spectral variability^28^. While high inter-subject variability could in theory be possible even in a high-quality data set (due to true physiological variations), the low variability suggests a well-standardized data set and animal model.
2. **Objectivity, reliability and validity of the HSI camera recordings**: Quality criteria of any research method are objectivity (extent to which the instrument is free from personal error or subjectivity on the part of the scorer and extent to which recordings remain interpretable retrospectively), reliability (extent to which the HSI measurements can be reproduced when repeatedly imaging the same sample) and validity (extent to which HSI measurements represent the real diffuse reflectance spectra). The camera operates at a fixed recommended distance to the target and acquisitions as well as retrospective evaluations are relatively operator-independent, suggesting objectivity of the method. In order to evaluate the reliability of the HSI camera, 13 consecutive recordings of the ColorChecker Classic® board from x-rite (Grand Rapids, Michigan, US), which is composed of 24 different standardized color chips, were taken with the HSI camera on two different dates. To assess validity of the HSI measurements, the spectral reflectances of the same colorchecker board were measured with a conventional point spectrometer (Ocean Insight HR2000 + ; light source: Tungsten Halogen lightsource Ocean Insight HL-2000 (formerly Ocean Optics; Orlando, Florida, US)). For each color chip, spectrometer measurements were conducted 100 times. To enable a comparison between spectrometer and HSI measurements, the spectrometer measurements were clipped to the spectral range 500 nm to 1000 nm and L1-normalization across the spectral dimension was performed as a preprocessing step for both the spectrometer and HSI data. The L1-normalized spectrometer spectra were further scaled by a factor of 11.31 to compensate for the difference in number of spectral channels (1131/100 channels between 500 nm and 1000 nm for spectrometer/HSI camera). Figure 7 illustrates for each color chip the median and standard deviation across all 100 normalized and scaled spectrometer measurements. For the HSI recordings, median spectra were computed over an area of 64 × 64 pixels for each color chip. These areas were annotated automatically over a homogenous color chip area with a safety margin to its borders. The annotations were always visually confirmed. The resulting annotation masks are included in the dataset. The median and standard deviation over the color chip median spectra of the 13 consecutive recordings are shown in Fig. 7. Consecutive HSI measurements are very similar with an average standard deviation below 0.0001. HSI measurements taken on different days are visibly similar. Agreement between spectrometer and HSI measurements is overall good with deviations occurring mainly in the near-infrared part of the spectrum. These deviations are most pronounced for color chips yielding small HSI intensities, such as dark skin, foliage and black. It can be concluded that reliability as well as validity of the HSI camera recordings are high.

**Fig. 8:**
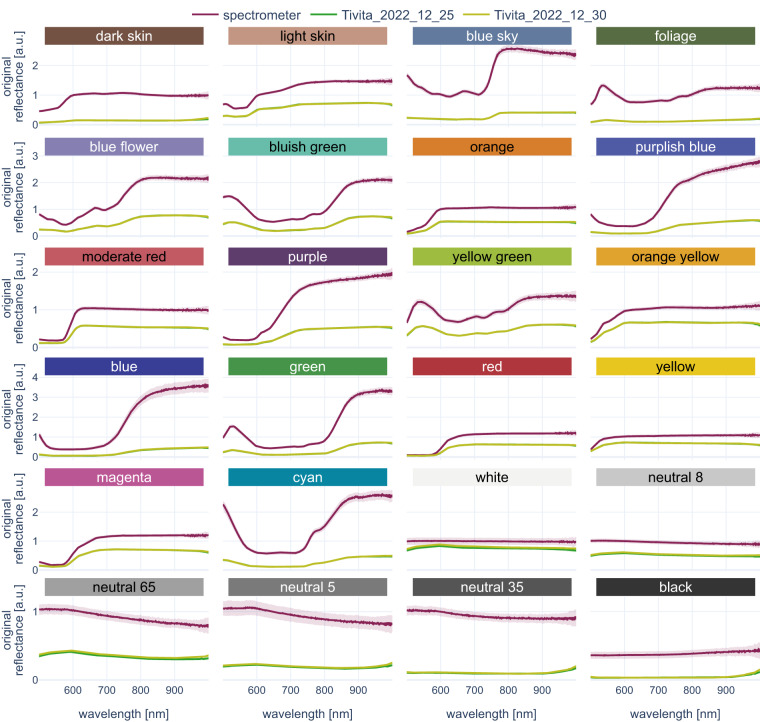
Original reflectance values of checkerboard-based technical validation show deviations. Spectrometer (purple) and HSI (light and dark green) recordings for the 24 different color chips. The median spectrum across all available recordings (100 recordings for spectrometer, 13 recordings for HSI) is denoted by a solid line, the corresponding standard deviation as a shaded area. An interactive version of this figure can be found and online (https://figures.heiporspectral.org/paper_figures/Figure_08_colour_checker_orig.html).
3. **Quality of the annotations**: The dataset was annotated by three independent experts (annotator 1–3). An annotation protocol ensured a standardized annotation procedure. However, as the annotations comprised the selection of a representative region of interest, intra- and inter-annotator variability cannot simply be measured by the overlap in annotated pixels. Instead, the quality was monitored and evaluated by cross-validation and by comparison of the extracted spectra. Note that while there were cases of great overlap of annotated regions (e.g. gallbladder; Fig. 9a), there were also cases in which annotated regions did not overlap at all (e.g. colon; Fig. 9b). To ensure that the region selection had a minor influence on the extracted spectra, spectral reflectance was visualized stratified by the three different annotators in Figs. 9, 10 and no substantial differences in spectral reflectance values were observed. For polygon boundary cross-validation, every annotation of the three annotators was validated by a medical expert assessor (with knowledge of the organ label) and there were no cases of disagreement regarding polygon boundaries. For assigned organ label cross-validation, we assessed the intra- and inter-annotator agreement (blinded for assigned organ label). The polygons of annotator 1 were reclassified by annotator 1 (intra-annotator) and an independent medical expert assessor (inter-annotator). An intra-annotator agreement of 100% could be achieved. The independent medical expert assessor achieved an inter-annotator agreement of 99.5% (27 of 5756 polygons were misclassified). All 27 conflicts could be resolved by consensus.

**Fig. 9:**
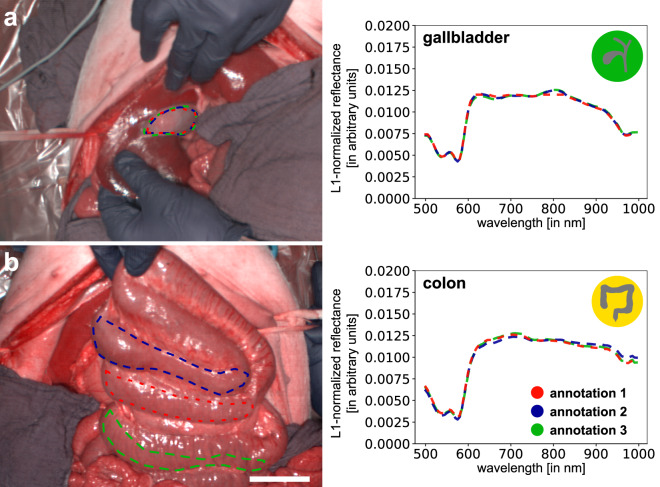
Annotations of three expert annotators for two exemplary HSI recordings. The three annotations are marked in red, blue and green. (**a**) great overlap of the annotations in the exemplary gallbladder recording. (**b**) no overlap of the annotations in the exemplary colon recording. In both cases, different annotators yield highly comparable spectral reflectance.

**Fig. 10:**
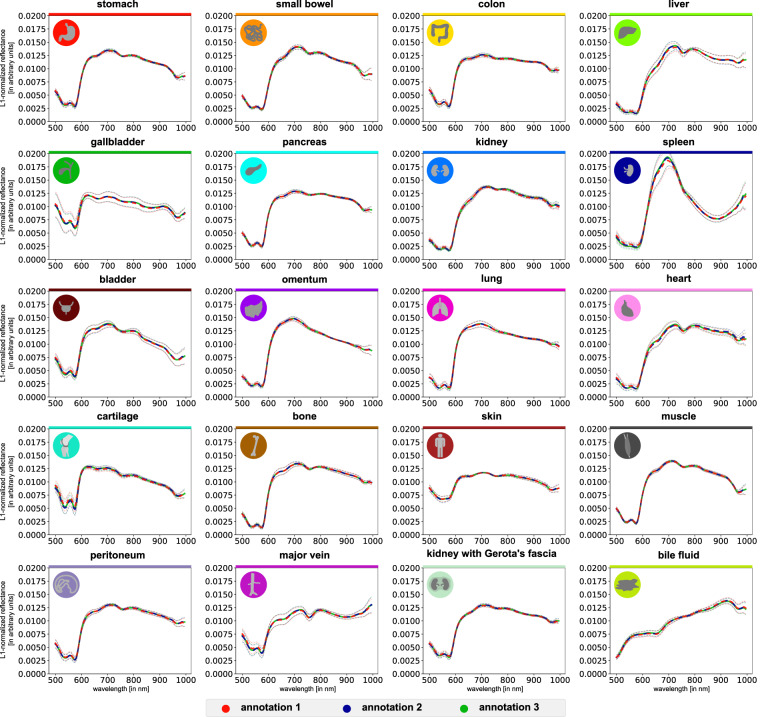
Spectral reflectances of single organs after L1-normalization are still highly similar across experts. Graphs depict mean reflectance (L1-normalized on pixel-level) of individual pigs (gray) as well as overall mean (blue) ±1 standard deviation (SD) (black) with wavelengths from 500 to 1000 nm on the x-axis and reflectance in arbitrary units on the y-axis.

## Usage Notes

The provided dataset is made available under the Open Data Commons Attribution License (CC-BY 4.0): https://creativecommons.org/licenses/by/4.0/. If readers wish to use or reference this dataset, they should cite this paper.

The dataset (https://www.heiporspectral.org/)^27^ contains the raw data as obtained from the HSI camera system, which includes the HSI cube and camera-related metadata (e.g. software version, exposure time) as well as screenshots of the tissue parameter images. Additionally, we publish annotation masks and precomputed files such as tissue parameter images, L1-normalized HSI cubes targeted at efficient data processing (e.g. for training neural networks), interactive visualizations for data exploration (https://figures.heiporspectral.org/paper_figures/) as well as aggregated tables of commonly used data (e.g. median spectra). Furthermore, reconstructed RGB images together with annotations and median spectra are available on the webpage (https://www.figures.heiporspectral.org/view_organs/). They can be used to gain an overview of the dataset and insights into individual images. To facilitate working with the dataset, we release the data repository together with a Python package (https://github.com/IMSY-DKFZ/htc) which can be used to iterate over the images in the dataset, selecting subsets of the data and functions to read the data cubes and the additional metadata. Tutorials and documentation are provided as entry points. Additionally, the package contains a complete pipeline for machine learning practitioners including highly efficient HSI data loading, training of various classification and segmentation networks and aggregation methods, which respect the hierarchy of the data for a correct and comparable validation as well as visualizations of the results. Finally, the package also contains pre-trained models (e.g. a U-Net-based organ segmentation network) published previously^29^.

## Data Availability

Data acquisition was performed with the TIVITA® Suite (version 1.6.0.1). The polygon annotations were created with a software developed in-house which is available on GitHub: https://github.com/MIC-Surgery-Heidelberg. All data analyses and visualizations in this manuscript were performed using Python and the corresponding code is available at https://github.com/IMSY-DKFZ/htc.
